# Lake water based isoscape in central-south Chile reflects meteoric water

**DOI:** 10.1038/s41598-021-87566-4

**Published:** 2021-04-22

**Authors:** Wesley P. Scott, Sergio Contreras, Gabriel J. Bowen, T. Elliott Arnold, Ramón Bustamante-Ortega, Josef P. Werne

**Affiliations:** 1 Department of Geology and Environmental Science, University of Pittsburgh, Pittsburgh, PA USA; 2 Departamento de Química Ambiental, Facultad de Ciencias & Centro de Investigación en Biodiversidad y Ambientes Sustentables (CIBAS), Universidad Católica de la Santísima Concepción, Casilla 297, Concepción, Chile; 3 Department of Geology and Geophysics, University of Utah, Salt Lake City, UT USA; 4 Centro de Información de Recursos Naturales (CIREN), Santiago, Chile

**Keywords:** Limnology, Climate sciences, Element cycles

## Abstract

Warming across the globe is expected to alter the strength and amount of regional precipitation, but there is uncertainty associated with the magnitude of these expected changes, and also how these changes in temperature and the hydrologic cycle will affect humans. For example, the climate in central-south Chile is projected to become significantly warmer and drier over the next several decades in response to anthropogenically driven warming, but these anthropogenic changes are superimposed on natural climate variability. The stable isotope composition of meteoric water provides significant information regarding the moisture source, pathways, and rain-out history of an air mass, but precipitation samples suitable for stable isotope measurements require long-term placement of field equipment making them difficult to obtain. The International Atomic Energy Agency (IAEA) Global Network of Isotopes in Precipitation (GNIP) stations generate isotopic and ancillary data of precipitation from many locations around the world, but remote areas of developing countries like Chile typically have sparse networks of meteorological stations, which inhibit our ability to accurately model regional precipitation. Central-south Chile, in particular, has a sparse network of GNIP stations and, as a result, the isotopic composition of meteoric water is underrepresented in the global database complicating efforts to constrain modern day hydroclimate variability as well as paleohydrologic reconstruction for southern South America. In this study, we measured the stable isotope compositions of hydrogen (δ^2^H) and oxygen (δ^18^O) in surface lacustrine waters of central-south Chile to determine what physical and/or climatic features are the dominant controls on lacustrine δ^18^O and δ^2^H composition, assess whether or not the isotopic composition of the lakes record time-averaged isotope composition of meteoric water, and determine whether an isoscape map based on lake surface waters could predict the H and O isotope compositions of precipitation at the few GNIP stations in the region.

## Introduction

The present-day hydroclimate on the western side of southern South America is dominated by the Southern Westerly Winds (SWW)^1^. The SWW are the strongest time-averaged winds on Earth and are tied to significant global climatic features, such as the Antarctic circumpolar current, sea surface temperature gradients in the southern Pacific Ocean, and CO_2_ exchange between the ocean and the atmosphere in the Southern Ocean^2^. The hydroclimate in central-south Chile is dominated by the SWW zonal flow, yielding a strong correlation between wind intensity and local precipitation. Recent studies have suggested that the core of the SWW is migrating poleward, significantly altering rainfall in the mid latitudes, but a full understanding of the influences of climate change on effective precipitation in the region remains elusive.

The stable isotope composition of H and O in water is a function of the cumulative isotope effects associated with conditions during evaporation over the open ocean and the rain-out history between initial formation of the water vapor and its ultimate deposition to the system of interest (e.g., lake water^3^). This relationship is expressed in the Global Meteoric Water Line (GMWL), which reflects the coupled, spatiotemporal variations in H and O isotope ratios of precipitation. The hydrogen (δ^2^H) and oxygen (δ^18^O) isotope values of lake water are integrated signals that can reflect local hydrologic processes such as precipitation and evaporation, and can also reveal physical landscape variations and other inputs to the lake such as groundwater, snow melt, and stream input^4^. Lake water δ^2^H and δ^18^O values reveal continental, regional, and local scale processes, ranging from climate and seasonality to individual watershed catchment features, such as watershed slope and altitude^5^.

Global isoscapes^6,7^ that interpolate precipitation isotope values between GNIP stations have been developed to give a spatial representation of isotopes in regions that lack an abundance of GNIP stations. While this development has been extremely powerful for studies involving precipitation isotopes, uncertainty of predicted isotope values increases in regions with sparse GNIP station coverage. For example typical uncertainties range from 20 to 40‰ for δ^2^H GNIP precipitation estimates in central Chile, rendering them ineffective for a clear picture of the isotopic composition of local precipitation. Thus, examining the δ^2^H and δ^18^O values of surface lacustrine water can be extremely useful as a proxy for present day precipitation δ^2^H and δ^18^O composition, which is crucial in areas that have existing lakes but little precipitation isotope data. Surface lake water data can then be used to calibrate hydrologic proxies that can aid in reconstructing a paleohydrologic record for a given lacustrine system. While many lacustrine systems fed by meteoric water are subject to isotopic enrichment through evaporation, which skews the isotopic signal away from the original meteoric source water^5^, others can record meteoric water signals with high fidelity.

**Figure 1:**
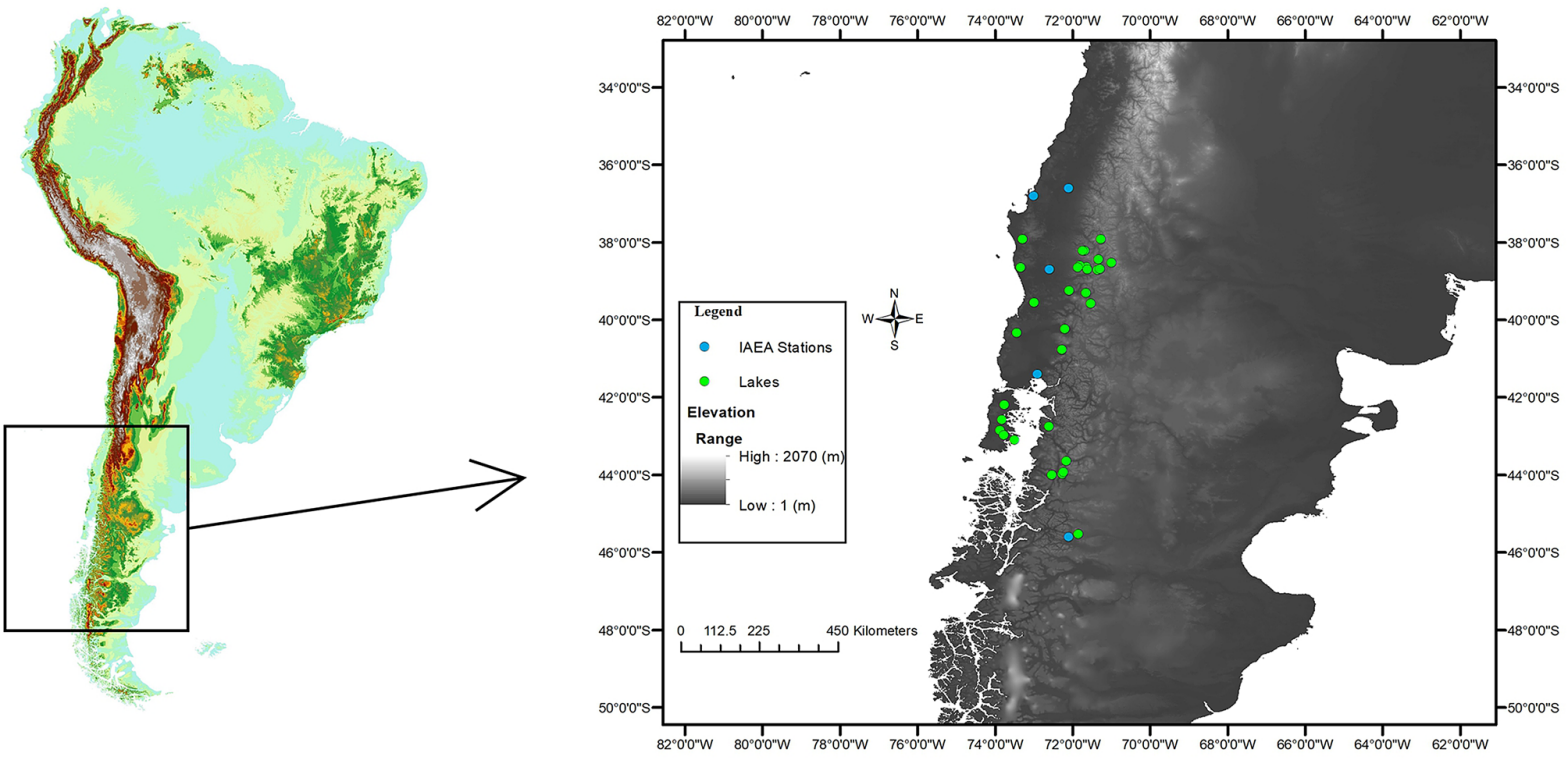
The region of study with lakes labeled with green circles and IAEA GNIP stations labeled with blue circles. Gray scale describes elevation. Map generated using QGIS^21^ software.

In southern Chile, large areas are spatially underrepresented by the meteorological stations currently in place, especially when it comes to monitoring the stable isotopes of local precipitation, leading to large gaps in data. Developing a more effective regional model is crucial for gaining a more complete and reliable data set for climate variability in this remote region. Here, we collected surface waters from 34 lakes spanning ~ 8 degrees of latitude and representing varying climate regimes with elevations ranging from near sea level to ~ 2000 m in central-south Chile (Fig. 1) for stable isotope analysis of hydrogen (δ^2^H) and oxygen (δ^18^O). We also collected data from the five IAEA GNIP stations in central-south Chile for statistical comparison and to better understand how isotopic values of meteoric water are transformed along the moisture pathway from source to deposition in coastal and alpine lakes. In addition, a multivariate analysis of the physical and climatic features of the region was developed in order to discover which features statistically have the most influence on the observed isotopic composition of the lacustrine waters, and to create a regional “isoscape”. Once the relationship between meteoric and lake water can be established, it is possible for the lakes to be used as proxies for time-averaged meteoric water. Lakes were delimited and drainage basins were described through utilization of open source QGIS software, and results indicate pristine conditions and open connectivity for most of the lakes, i.e. there are both inflowing and outflowing streams at each site.

### Study site

The lakes included in this study span approximately 38°S–46°S in Chile, South America, a region that includes two well-defined climate regions^8^ that transition from a warmer and drier Mediterranean climate (Csb on the Koppen classification scheme) in the northern area of study to a cooler and wetter temperate oceanic (Cfc) climate in the south (Fig. 1). Central Chile is characterized by a temperate climate with significant seasonality that includes a considerable amount of precipitation, usually concentrated in the austral winter (JJA), with a clear seasonality in precipitation that is lost further south with colder and rainy (up to 5000 mm/yr) weather becoming more prevalent. Lakes in this study also span a wide vegetation gradient from north to south, changing from sclerophyllous forest to deciduous forest, broadleaved forest and evergreen forest^9^. The coastal mountain range (maximum elevation ~ 700 m, 73°W–74°W) is the first significant feature of elevation that induces precipitation. Moving eastward, the air parcels travel over the central Chilean valley and eventually encounter the Andes mountains (71°W–72°W), where significant rainout resulting from the orographic effect occurs, producing some of the highest mean annual precipitation (MAP) values of this study region. This study represents 34 of the approximately 90 lakes in the region, providing fair representation where many of the lakes are extremely remote and difficult to access.

During austral summer (DJF), the core of the SWW lies south of 50°S, and the SWW delivers large amounts of rainfall south of 48° during this interval^2^. During the austral winter (JJA), the SWW migrates to the north, and winter rainfall provides most of the total annual rainfall to our study region. Since 2010, central Chile has experienced what has been referred to as a “mega-drought,” which has significantly influenced water availability^10^ and the frequency of regional forest fires. This has been attributed to anticyclonic and cyclonic anomalies over the South Pacific and the Amundsen and Bellingshausen Seas resulting in the alteration of mid-latitude storm tracks^11^. It has been considered the longest and driest period of record in this region, and climate models suggest that this dry trend will continue to occur for the foreseeable future^12^. In addition to seasonality and sea surface temperatures in the South Pacific, the amount and timing of precipitation is also affected by interannual variability related to El Niño/Southern Oscillation (ENSO). During years of El Niño, 30°S–35°S experiences an increase in rainfall during the winter months, 35°S–38°S receives an increase in rainfall in the spring, and 38°S–41°S receives less rainfall during the summer months^13^.

Significant effort has been put into studying modern day climate in Patagonia, south of 42°. For example, H and O isotopes were measured in streams between ca. 40°S to 48°S^14^. The authors suggest that topography is the dominant control on the isotopic composition of meteoric and surface waters in Patagonia (47°S–48°S), and used deuterium excess (*d*) as a measure of the amount of evaporation in the surface waters, where lower *d* values (< 5‰) indicate large amounts of evaporation. Streamwater samples from the west show typical δ^2^H and δ^18^O values of − 30‰ and − 4‰ respectively, while samples from the east are isotopically depleted, with δ^2^H and δ^18^O values of − 110‰ and − 14‰, respectively^15^. Here the authors suggested that stream samples are representative of long term averages of precipitation δ^2^H and δ^18^O values in Patagonia. The local evaporation line generated by these authors bears a similar slope and intercept to the GMWL (and the present study, see below), and also provides significant spatial representation. However, north of 40°S where the edge of the SWW reaches its northernmost extent, data is lacking. Additionally, while stream water may in fact be representative of meteoric water, streams do not offer the sedimentological archive that lakes provide, limiting their usefulness for paleohydroclimatic studies. Here, we provide stable isotopic data for more than 30 lakes in southern South America, in an effort to determine the significant geographic and climatic controls on lake water isotopic composition and create a lake “isoscape” that is significant in a region with sparse hydrologic data. Lacustrine isotopic composition is widely used in paleoclimatic studies^16–18^, and because the isotopic composition of ancient meteoric water is unknown, lakes provide a reasonable modern day baseline that allows for the interpretation of the effects of current climatic and physical conditions.

## Materials and methods

Lakes were selected based on location, accessibility, and elevation throughout central to southern Chile to provide a range of physical attributes that have been shown to be significant controls on meteoric water isotopic composition^19^. Lake water was collected over a three year period from 2017 to 2019 in spring and summer campaigns, several lakes were sampled in multiple years (supplementary Table 1). Samples (30 ml) were taken from the center of each lake at ~ 1 m water depth in opaque Nalgene bottles, and stored refrigerated until analysis. Water samples were then transferred to 2 mL glass vials using a sterile glass Mohr pipette for each sample.

The H and O isotope composition of water samples was measured using an integrated cavity ring-down spectroscopic analyzer coupled to a liquid water vaporizer (Los Gatos Research Inc.). The vaporizer temperature and gas pressure were ~ 43℃ and ~ 155 torr, respectively. All isotope values reported are in conventional delta notation (as per mil (‰) deviations from a standard, where ${\delta }^{2}H =(( (D/H_{sample})/(D/H _{standard}))-1)*1000$. Calibration was performed with each run using USGS water isotope standards, and measurements were standardized to the VSMOW scale. Analytical precision (1σ) based on eight replicate sample measurements averaged ~ 1‰ for δ^2^H and under 0.5‰ for δ^18^O, with eight replicate injections for each sample/standard. Memory effects between samples were evaluated with standard measurements. Six of eight sample injections of each sample were used for uncertainty calculations. A One-way Analysis of Covariance (ANCOVA) was conducted to determine statistically significant differences between slopes of the LMWL and GMWL.

Lakes were plotted on a digital elevation model (DEM) of South America using ArcMap (Fig. 1). The lake watersheds were delimited over a DEM of 12 m resolution sourced from the Japanese Aerospace Exploration Agency (JAXA^20^). Open source software QGIS^21^ was used to identify lake inlets/outlets over simulated hydrology and hill shade model calculated from the DEM. The watershed was then delimited, with corrections made to better define the edges of each polygon used to calculate boundaries, such as erasing small polygons that were interpreted as noise. Slope was also calculated according to methods/ranges proposed by the United States Department of Agriculture^22^ so that each watershed is described with a slope characteristic, with slope gradient classes above 45 indicating steep drainage basins. The land uses were calculated from Chilean vegetative data^20^.

A model selection procedure was used to develop an optimized model describing the spatial variation in multi-year average (unweighted) lake water isotope values. Geographic and climatological variables, including elevation, latitude, longitude, mean annual air temperature (MAT) and MAP were used and a stepwise selection was conducted considering all variables and their first order interactions. MAT and MAP were calculated from a new climatological dataset available online (CR2MET) from a monthly reanalysis product with a 0.05° horizontal resolution considering the period from 1979 to 2016, extracting data from the nearest grid point to the lake positions where we collected surface water. Elevation was obtained from the United States Geological Survey^23^. Precipitation isotope data were collected from the IAEA database^24^ and long-term (the length and temporal resolution of each station record varied greatly) mean-annual isotope ratios, weighted by monthly precipitation amount, were calculated for each of the five monitoring sites. Models were compared using ANOVA and the AIC statistic. Because of the strong coupling between H and O isotope ratios, we modeled the multivariate response of both isotope systems. The optimal model was parameterized in terms of longitude, latitude, and MAP, and explains 80% of the variance in δ^18^O and 90% of the variance in δ^2^H values. Two outliers with low deuterium excess (< 5‰) were removed from the analysis; these were identified as outliers in the model diagnostics, as well. The optimized model and gridded MAP was used to develop prediction maps for lake water isotope distributions at 0.05 degrees resolution. All statistical calculations were conducted in the R programming environment^25^.

## Results

**Figure 2:**
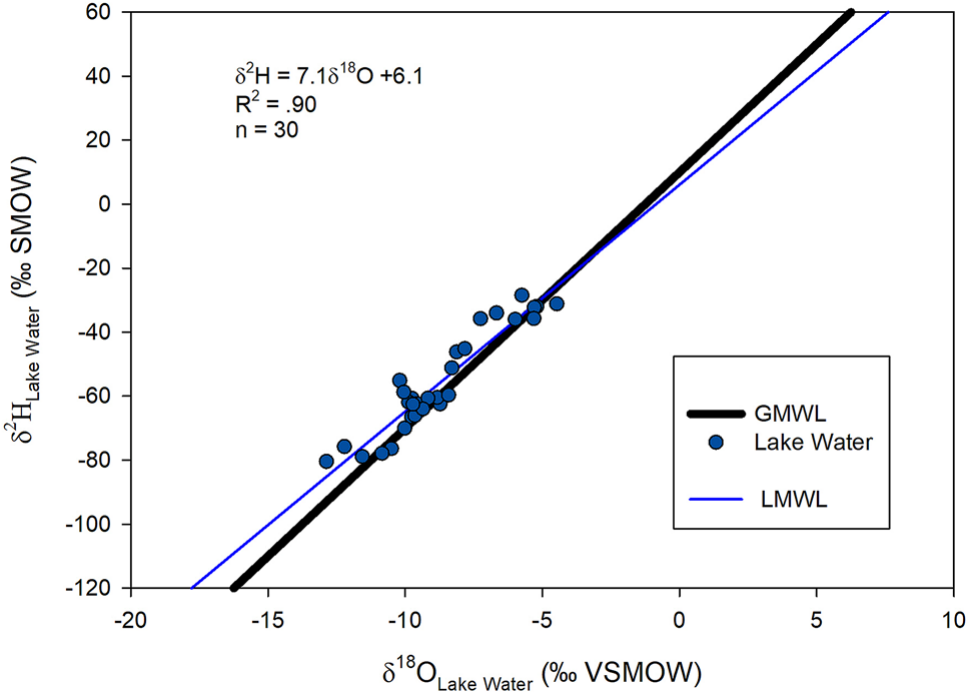
The GMWL (black line) and lake water δ^2^H and δ^18^O values plotted (blue circles) and the resulting LMWL (blue). Data represent averages of multiple measurements taking in different sampling years (up to n = 3) where possible.

δ^2^H values of the lakes range from approximately -80‰ to -23‰ and δ^18^O values range from -12‰ to -4‰ (Fig. 2, Table 1). These values are comparable with stream water isotope values determined by Stern and Blisniuk^14^ and Smith and Evans^15^ and are also similar to a study of lakes in the state of Oregon, USA. We were able to get two or three years of consecutive sampling for 15 of the lakes, and the standard deviation of these replicate values for individual lakes was small, with an average of 2.8‰ and 0.92‰ for δ^2^H and δ^18^O, respectively (supplementary Table 1). A plot of δ^2^H vs. δ^18^O yields a strong linear correlation (r^2^ = 0.90) and a resulting linear equation of δ^2^H = 7.1 $*$ δ^18^O + 6.1 that closely mimics the GMWL equation (δ^2^H = 8 $*$ δ^18^O + 10). In fact, no significant differences between slopes were found comparing LMWL and GMWL (p < 0.05). For the generation of the local meteoric water line (LMWL), the three collection years were averaged to eliminate any anomalous time periods that would not be representative of long term conditions of the lakes.

**Table 1:** Lakes with physical, climatological, and isotopic data from this study.

Lake name	Latitude (°S)	Longitude (°W)	Surface area (km^2^)	Mean elevation (m.a.s.l)	Slope characteristics	Open/closed	Connectivity to other lakes	% Snow and glaciers	MAT (°C)	MAP (mm/year)	Averaged δ^2^H (‰ VSMOW)	Averaged δ^18^O (‰ VSMOW)
El Barco	37.923379	71.276323	44.3	1768	29	Open	–	0.0	7.7	1905	− 75.8	− 12.2
Lanalhue	37.920092	73.289833	356.0	335	28	Open	–	0.0	12.6	1101	− 28.5	− 5.7
Verde Tolhuaca	38.214000	71.734000	1.8	1521	39	Open	Outflow to Malleco	17.5	7.0	2760	− 64.0	− 9.4
Malleco	38.216200	71.821500	44.5	1250	39	Open	Inflow from Agua De Verde	6.7	7.5	2882	− 62.4	− 9.6
San Pedro	38.441521	71.333549	0.0	913	3	Open	–	0.0	8.4	2196	− 62.9	− 6.6
Verde (Pehuenco)	38.520000	70.992000	0.2	1834	26	Open	–	0.0	6.2	1973	− 80.4	− 12.9
Negra	38.587454	71.810661	38.3	1200	19	Open	–	0.0	9.5	2471	− 60.7	− 9.2
Conguillio	38.632800	71.640300	50.0	1388	28	Open	Outflow to Verde PN	0.0	7.2	2752	− 70.5	− 9.3
Quepe	38.649129	71.868643	32.7	1108	23	Open	–	5.3	8.6	2376	− 59.7	− 8.4
Trovolhue	38.650600	73.341300	5.3	108	14	Open	–	0.0	–	–	− 34.0	− 6.7
Galletue	38.679700	71.287200	217.0	1434	27	Open	Inflow from El Toro	0.0	11.7	2218	− 76.4	− 10.5
Captren	38.639900	71.702210	2.5	1365	21	Open	–	0.0	7.6	2877	− 61.9	− 9.9
Verde Conguillio	38.684360	71.610075	140.1	1396	33	Open	Inflow from Conguillo	3.2	8.2	2428	− 70.0	− 10.0
El Toro	38.708079	71.349900	98.1	1498	32	Open	Outflow to Galletue	0.0	7.4	2462	− 62.5	− 8.7
Villarrica	39.242500	72.092500	1488.7	803	27	Open	Inflow from San Jorge	0.0	11.4	2000	− 62.6	− 9.7
San Jorge	39.309540	71.651680	9.2	1186	38	Open	Outflow to Villarrica	0.0	–	–	− 58.8	− 10.1
Escondida	39.574400	71.529200	2.7	1361	40	Open	Outflow to Quillehue	0.0	6.8	3259	− 60.5	− 8.8
Coipolafken	40.239850	72.194880	0.2	418	18	Open	–	0.0	10.3	3041	− 46.2	− 8.1
Trinidad	40.339900	73.438800	105.1	285	25	Open	–	0.0	–	–	− 35.8	− 7.3
Toro (Puy)	40.769480	72.269030	5.2	860	28	Open	–	0.1	8.5	3253	− 55.1	− 10.2
Cajunco	42.194700	73.761300	8.7	134	12	Open	–	0.0	10.8	1671	− 32.0	− 5.2
Millan de Canaan	42.583500	73.822800	2.5	107	6	Open	–	0.0	10.7	1513	− 31.2	− 4.5
Blanco	42.748500	72.609700	93.9	732	40	Open	–	0.0	8.7	4813	− 51.2	− 8.2
Rinihue	42.846400	73.872900	5.3	148	7	Open	–	0.0	10.4	1580	− 36.0	− 6.0
NN Tantauco	42.975500	73.773800	9.1	217	10	Open	–	0.0	10.2	1730	− 35.7	− 5.3
Cipreces	43.099800	73.501100	2.3	42	6	Open	–	0.0	10.8	2054	− 32.3	− 5.3
Laguna Negra	43.649800	72.156800	12.5	600	41	Open	–	0.0	8.2	3462	− 78.9	− 11.6
Claro del Solar	43.935700	72.237900	594.5	988	56	Open	–	0.0	8.0	2564	− 77.9	− 10.8
Negro	43.974000	72.265700	14.1	350	37	Open	–	0.0	8.0	2569	− 66.5	− 9.8
Berger	44.009400	72.531400	0.5	125	27	Open	–	9.7	8.8	2938	− 60.7	− 9.7
Toro (Coy) A	45.531424	71.854938	0.0	717	19	Open	–	1.1	8.5	807	− 64.9	− 4.9

δ^2^H values have the strongest positive linear correlation with longitude of the lake site (r^2^ = 0.77, see supplementary Fig. 1). A positive linear relationship with δ^2^H also occurs with mean annual temperature (MAT) (r^2^ = 0.49, see supplementary Fig. 2) of the lake site. δ^2^H has a negative linear correlation with lake elevation (r^2^ = 0.37), and a weak linear relationship with latitude (r^2^ = 0.10) and MAP (r^2^ = 0.26). The only strong positive linear correlation that is revealed between δ^18^O values and the model parameters was with longitude (r^2^ = 0.54, see supplementary Fig. 3). δ^18^O does not correlate well with elevation or MAT (r^2^ = 0.26 and r^2^ = 0.32, respectively). MAT obtained from higher (0.05°) resolution monthly reanalysis show improved fit with in situ MAT data (data not shown) compared to MAT estimated from default reanalysis (0.5° resolution^26^). Values of deuterium excess (*d*, defined as *d* = δ^2^H – 8 $*$ δ^18^O, not shown)*,* ranged from 4 to 26‰, with two outliers with negative *d* values that were excluded from the linear regression models.

**Figure 3:**
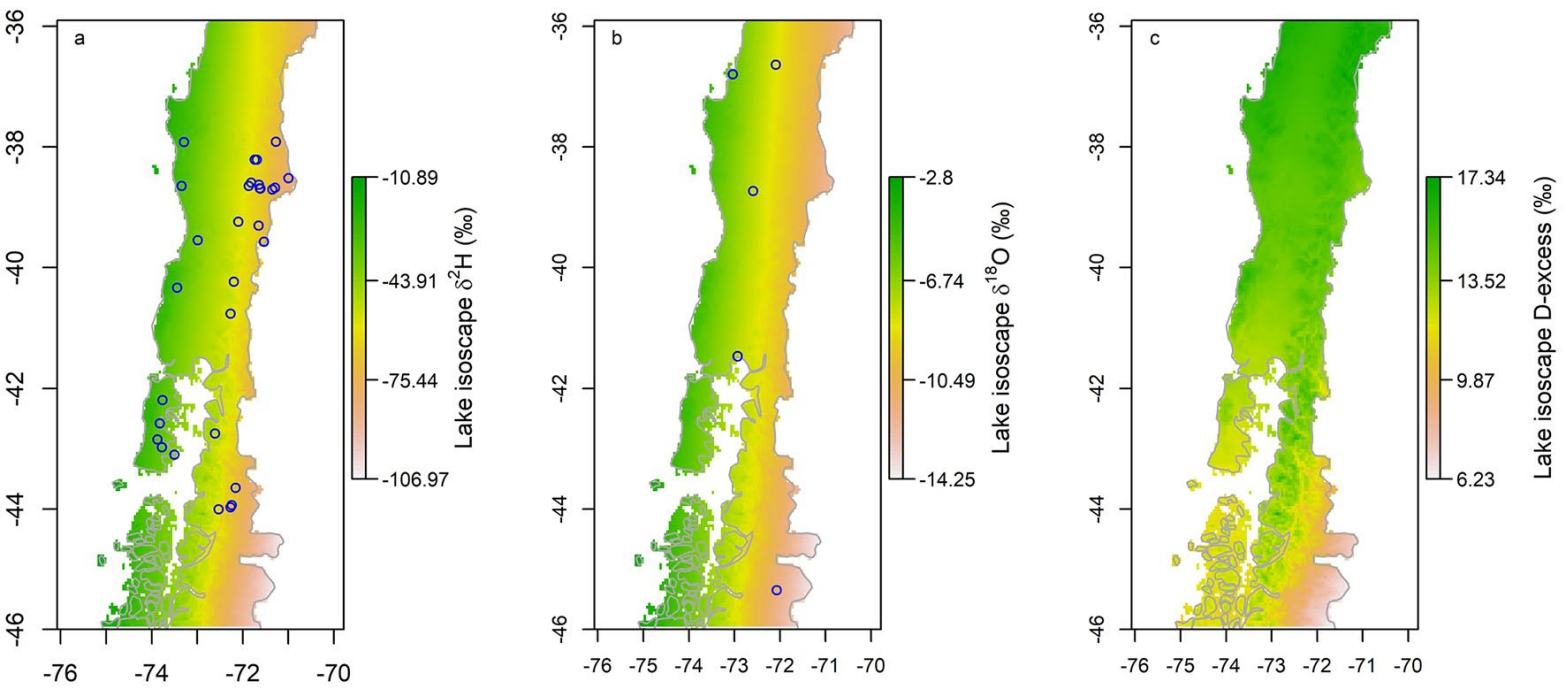
(**a**) Multivariate isoscape model predicted δ^2^H (color scale) for our study region, based on isotope values measured at lake locations (circles) (**b**) Predicted δ^18^O (color scale) and IAEA GNIP station locations (circles) (**c**) Predicted deuterium excess (color scale), all at 0.05° resolution. Map generated using QGIS^21^ software.

**Figure 4:**
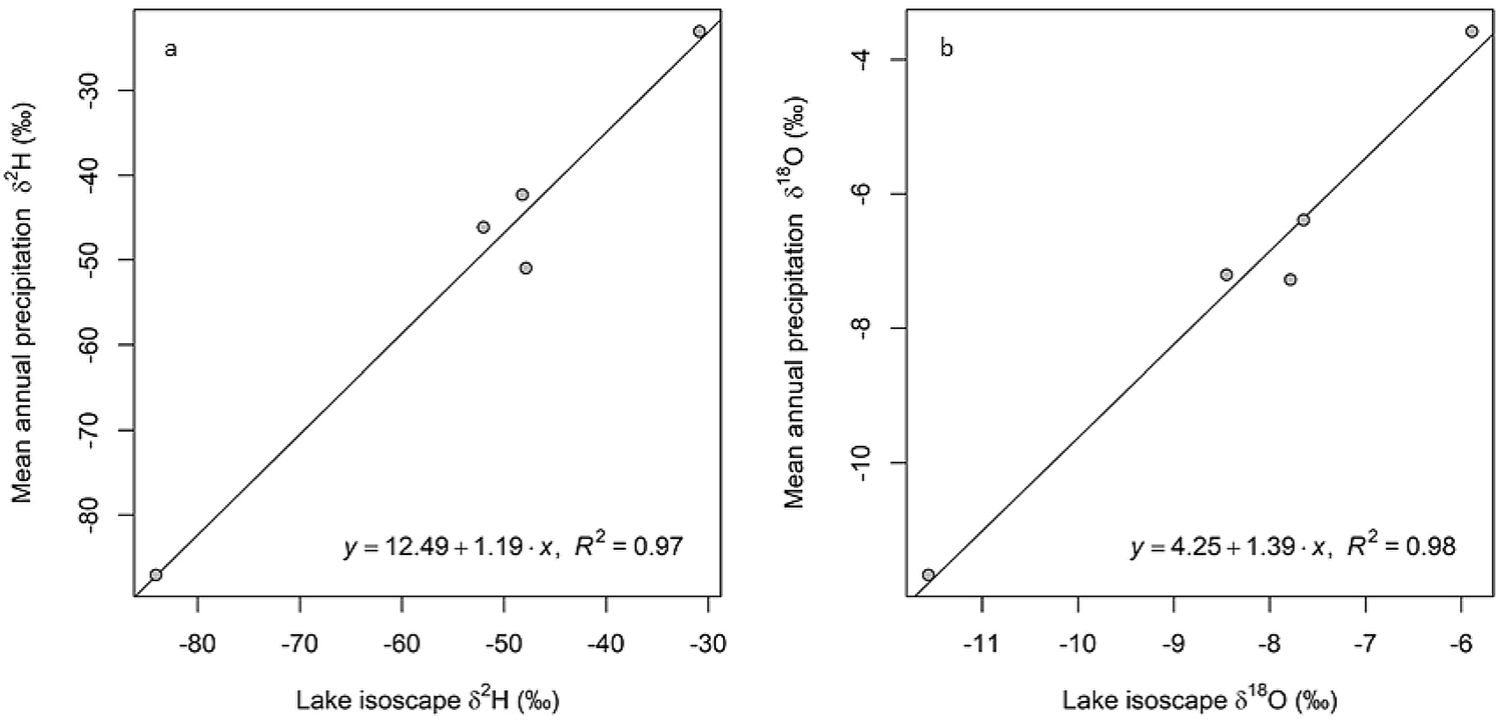
δ^2^H values from GNIP data plotted vs. model predicted δ^2^H values (**a**), δ^18^O values from GNIP data plotted vs. model predicted δ^18^O values (**b**).

The results of the multivariate regression model can be seen in Fig. 3, and predict approximately 80% of the variance in lake water δ^18^O and 90% of lake water δ^2^H. Of the three variables included in the multivariate analysis, longitude explained most of the variance, followed by latitude and MAP, respectively. The equations for δ^2^H and δ^18^O generated by the model are as follows: ${\delta }^{2}H=3.865*(Latitude)-22.74*(Longitude)+3.938*{10}^{-3}*(MAP)-1.554*{10}^{3}$ and ${\delta }^{18}O=0.373*(Latitude)-2.74*(Longitude)+3.456*{10}^{-4}*(MAP)-1.928*{10}^{2}$. Within the region of study, there are five IAEA GNIP stations that report monthly stable isotope values in precipitation that were used for comparison of our isoscape generated values. Isoscape-estimated lake-water δ^2^H and δ^18^O values at the location of these stations were strongly positively correlated with GNIP precipitation-weighted mean δ^2^H and δ^18^O (R^2^ = 0.97 and R^2^ = 0.98, respectively, Fig. 4a,b).

Lake watershed delimiting and classification reveals that all lakes in this study are hydrologically open. A slope index percentage was generated to assess the average steepness of each drainage area, following a previously determined guideline^22^, where percentages indicate terrain as follows: 0–3% is flat, 3–8% is undulating, 8–15% is moderately sloping, 15–30% is hilly, 30–45% is moderately steep, 45–65% is steep, and > 65% is very steep. The average slope of the lakes is 26%, or hilly. Most lake drainage areas are covered with forest and have little urban or agricultural land use (Table 1), indicating the remote and pristine nature of all lakes but two which are statistical outliers. Presence of snow or glacial coverage was found only in six lakes, all of which have less than 20% snow/glacial coverage of the total surface area. The average lake surface area is around 100 km^2^ although many of the lakes are well under 50 km^2^. Some lakes were also found to have connectivity to other lakes in this study, as indicated in Table 1.

## Discussion

The majority of the precipitation in this study region originates from mid-latitude cyclones and storm systems in the southern Pacific Ocean^27^. The SWW then push the moisture-laden air masses from west-to-east across southern South America, which are uplifted as they encounter the Andes Mountains, which is reflected in the significant relationship between longitude and lake water δ^2^H and δ^18^O values in both the raw isotope and model predicted values. In this region, longitude serves as a measure of distance from the coast and moisture source, and reveals the Rayleigh or rain-out effect on precipitation that is progressively raining out heavier isotopologues as the water masses move across the continent. Because both the coastal and Andes Mountains in central-south Chile are meridional, longitude is roughly representative of elevation, though we note that in our study area elevation is higher in the northern section, and there is also a greater distance from the coast to the mountains in the northern section, both of which likely reduce the correlations between δ^18^O and other parameters. Additionally, longitude and MAP are closely related because the MAT decreases as the altitude increases from west to east across the Andes Mountains, resulting in enhanced rainout as the air masses encounter elevation. The multivariate model also reflected these physical features of the study area, where longitude was the most influential on lake water isotopes, followed by latitude and MAP, respectively. The relationship with latitude is not as clear cut, possibly because most of the lakes sampled at lower latitudes are located at higher elevations in the Andes Mountains, and likely receive input from precipitation that is isotopically depleted.

We did not observe an evaporative enrichment signal in our lake water or modeled isotope values. This is not surprising because our study area is characterized mainly by forest land use with high average humidity and high MAP (Table 1), which does not favor strong evaporative conditions. Additionally, low-altitude moisture such as persistent fog and more frequent precipitation could be providing these watersheds with a more regular, albeit small, supply of meteoric water^28^, though more work is needed to examine the effects of fog and low-altitude moisture input. Most of the lakes are located either in the foothills or in the Andes Mountains which are likely receiving inputs from snowmelt and alpine streamwater. However, as shown by Stern and Blisniuk^14^, Patagonian streams represent a time-averaged meteoric water isotopic signature, so we expect this would not significantly alter the lake water isotope signature. Therefore, the δ^2^H and δ^18^O values of lake water are most likely influenced by a combination of several factors, but appear to reflect meteoric water with high fidelity. Indeed, comparison of our data with available GNIP isotope data, the lake water isotope compositions are similar to those of local precipitation (Fig. 4), although the lake water tends to be slightly depleted in ^2^H and ^18^O isotopes relative to local precipitation from GNIP stations. Many of the study lakes are located near the coastal mountain range or the Andes mountain range, and may be receiving some inputs from higher elevation source areas where precipitation is typically depleted in ^2^H and ^18^O relative to lower elevation areas. After the lake watersheds were delimited (Table 1) and analyzed for general characteristics such as vegetation cover, percent coverage of snow/glaciers, and connectivity, no single contributing factor is clear that leads to a signal that is slightly isotopically depleted relative to local meteoric water. Groundwater input could also be influencing the isotopic signal, as it has been widely found that the isotopic composition of groundwater represents a time-averaged meteoric water signal.

High *d* has been attributed to local water vapor recycling in which evaporated lake water vapor mixes with continental atmospheric water vapor leading to high *d* in water vapor above the lake in systems located in Madagascar^29^, the Laurentian Great Lakes^30^, and the Amazon^31^. Moisture recycling in southern Chile does not seem likely, however, because the supply of meteoric water comes directly from the southern Pacific Ocean and is rained out into the lakes studied without traveling a substantial distance over the continent. The average *d* of lakes in this study is 4‰ higher than the global meteoric average, once again suggesting the possibility of other inputs into the lake affecting the isotopic composition. In addition to moisture recycling, physical conditions at the source determine the *d* values of the water vapor evaporated from the open ocean. Conditions that enable rapid evaporation, such as low relative humidity (RH) and high wind speed can lead to high *d* in resulting precipitation^32^. During austral winter, when most of the precipitation occurs in the northern sector of this study, mid-latitude cyclones off the western coast of southern Chile in the southern Pacific Ocean have large regions of low relative humidity and extremely high wind speeds above the ocean and generate a significant amount of precipitation for central-south Chile^33^. Although SST is relatively low in the southern Pacific Ocean, the combination of low RH and high wind speeds at the moisture source regions creates favorable conditions for high *d* in the resulting meteoric water that is delivered to central-south Chile.

The lake isoscape model produced in this study identified the primary physical controls on lake water isotope composition, explaining 80–90% of the variance. However, perhaps more important is the demonstrated ability to predict the isotopic composition of lake water in areas where there is no physical precipitation data available. The model showed that the lakes studied are essentially reservoirs of meteoric water, and the lake isoscape now enables a much higher resolution model for predicting meteoric water isotopic composition compared to previously developed online isoscape models^7^. The isoscape developed based on these lakes indicates that, at least in some regions, such models based on lake water can be used as an analogue for time averaged local precipitation isotope composition in regions where meteoric isotope data are scarce. This novel approach is much needed in regions that are understudied, and typically have few, if any, long term monitoring stations. Central-south Chile is an area where hydrologic deficits are becoming more prolonged and intense, with hydrological deficit reaching even Valdivia (39.8°S), where rainforest is the original vegetation^1^. In addition, paleoclimatic reconstructions of the region are of interest due to the climatic significance of the SWW on both a local and global scale, and would benefit from a modern regional baseline within which to interpret paleodata, which this isoscape provides. Indeed, such an approach is relevant not just to our study region but could be applied to lake waters in selected systems worldwide that primarily record meteoric water, providing another tool to enhance understanding of climate variability.

## Conclusions

Although there is significant seasonality in this region, the fact that our data produce a LMWL that is statistically similar to the GMWL and strongly correlate with GNIP precipitation isotope values indicates that lake surface waters in this region represent an integrated isotopic signal of local meteoric water. This observation is consistent with other studies that have shown that coastal lakes often plot on or near the GMWL^34^. Although only approximately 50% of the lakes in this study are truly coastal, the group of lakes as a whole are representative of the meteoric water isotopic composition with possible contributions from relatively depleted high altitude sources, which could explain why the lake water isotopic compositions are slightly offset relative to GNIP precipitation. The lack of an enrichment signal from evaporation within the lakes is likely the result of a combination of several environmental factors, including coastal geographic locations, environmental conditions around the lakes, and hydrologic inputs to the lakes with lower δ^2^H and δ^18^O values (e.g. high elevation snowmelt or groundwater). Slightly elevated deuterium excess can be attributed to environmental conditions in the source region of the water vapor raining out over the study region. The isoscape model applied using lake water rather than precipitation isotope values demonstrates the potential to develop higher resolution isoscapes in regions with little monitoring data, thereby providing a new tool with which to explore remote and understudied regions globally.

## Supplementary Information

Supplementary Table 1.

Supplementary Figures.

## Data Availability

All data are available in Pangaea database (www.pangaea.de).
