# Author Correction: Lake water based isoscape in central-south Chile reflects meteoric water

**DOI:** 10.1038/s41598-021-98264-6

**Published:** 2021-09-16

**Authors:** Wesley P. Scott, Sergio Contreras, Gabriel J. Bowen, T. Elliott Arnold, Ramón Bustamante‑Ortega, Josef P. Werne

**Affiliations:** 1 Department of Geology and Environmental Science, University of Pittsburgh, Pittsburgh, PA USA; 2 Departamento de Química Ambiental, Facultad de Ciencias & Centro de Investigación en Biodiversidad y Ambientes Sustentables (CIBAS), Universidad Católica de la Santísima Concepción, Casilla 297, Concepción, Chile; 3 Department of Geology and Geophysics, University of Utah, Salt Lake City, UT USA; 4 Centro de Información de Recursos Naturales (CIREN), Santiago, Chile

Correction to: *Scientific Reports* https://doi.org/10.1038/s41598-021-87566-4, published online 22 April 2021

The original version of this Article contained errors in Table 1 where the commas in “Latitude” and “Longitude” were misplaced and some stable isotope data were incorrectly given. The original Table 1 and accompanying legends appear below.

**Table 1:** Lakes with physical, climatological, and isotopic data from this study.

Lake name	Latitude (°S)	Longitude (°W)	Surface area (km^2^)	Mean elevation (m.a.s.l)	Slope characteristic	Open/closed	Connectivity to other lakes	% Snow and glaciers	MAT (°C)	MAP (mm/year)	Averaged δ^2^H (‰ VSMOW)	Averaged δ^18^O (‰ VSMOW)
El Barco	37,91667	71,26667	44,3	1768	29	Open	–	0,0	7,7	1905	− 75,8	− 12,2
Lanalhue	37,92009	73,28983	356,0	335	28	Open	–	0,0	12,6	1101	− 28,5	− 5,7
Verde (Tol)	38,21400	71,73400	1,8	1521	39	Open	Outflow to Malleco	17,5	7,0	2760	− 66,4	− 9,0
Malleco	38,21515	71,68774	44,5	1250	39	Open	Inflow from Agua De Verde	6,7	7,5	2882	− 65,2	− 9,1
San Pedro	38,44152	71,33355	0,0	913	3	Open	–	0,0	8,4	2196	− 67,5	− 6,8
Verde (Pehuenco)	38,52000	70,99200	0,2	1834	26	Open	–	0,0	6,2	1973	− 80,4	− 12,9
Negra PN Cong	38,59420	71,81080	38,3	1200	19	Open	–	0,0	9,5	2471	− 59,5	− 7,1
Conguillio	38,63280	71,64030	50,0	1388	28	Open	Outflow to Verde PN	0,0	7,2	2752	− 70,5	− 9,3
Quepe	38,65000	71,86500	32,7	1108	23	Open	–	5,3	8,6	2376	− 60,7	− 7,7
Trovolhue	38,65060	73,34130	5,3	108	14	Open	–	0,0	–	–	− 34,0	− 6,7
Galletue	38,67970	71,28720	217,0	1434	27	Open	Inflow from El Toro	0,0	11,7	2218	− 78,0	− 10,3
Captrn	38,68936	71,62317	2,5	1365	21	Open	–	0,0	7,6	2877	− 64,6	− 9,2
Verde PN Cong	38,69470	71,61140	140,1	1396	33	Open	Inflow from Conguillo	3,2	8,2	2428	− 73,7	− 9,5
El Toro	38,70808	71,34992	98,1	1498	32	Open	Outflow to Galletue	0,0	7,4	2462	− 62,5	− 8,7
Villarrica	39,24250	72,09250	1488,7	803	27	Open	Inflow from San Jorge	0,0	11,4	2000	− 62,6	− 9,7
San Jorge	39,30954	71,65168	9,2	1186	38	Open	Outflow to Villarrica	0,0	–	–	− 58,8	− 10,1
Escondida	39,57440	71,52920	2,7	1361	40	Open	OUTFLOW TO QUILLEHUE	0,0	6,8	3259	− 60,5	− 8,8
Coipolafken	40,23985	72,19488	0,2	418	18	Open	–	0,0	10,3	3041	− 46,2	− 8,1
Trinidad	40,33990	73,43880	105,1	285	25	Open	–	0,0	–	–	− 35,8	− 7,3
Toro (Puy)	40,76948	72,26903	5,2	860	28	Open	–	0,1	8,5	3253	− 55,1	− 10,2
L. Cajunco	42,19470	73,76130	8,7	134	12	Open	–	0,0	10,8	1671	− 32,0	− 4,6
Lago Millan de Canaan	42,58350	73,82280	2,5	107	6	Open	–	0,0	10,7	1513	− 31,2	− 4,5
L. Blanco	42,74850	72,60970	93,9	732	40	Open	–	0,0	8,7	4813	− 51,2	− 8,2
L. Renihue	42,84640	73,87290	5,3	148	7	Open	–	0,0	10,4	1580	− 36,0	− 6,0
L. NN Tantauco	42,97550	73,77380	9,1	217	10	Open	–	0,0	10,2	1730	− 35,7	− 5,3
L. Cipreces	43,09980	73,50110	2,3	42	6	Open	–	0,0	10,8	2054	− 32,3	− 5,3
L. Negra	43,64980	72,15680	12,5	600	41	Open	–	0,0	8,2	3462	− 78,9	− 11,6
L. Claro del Solar	43,93570	72,23790	594,5	988	56	Open	–	0,0	8,0	2564	− 77,9	− 10,8
L. Negro	43,97400	72,26570	14,1	350	37	Open	–	0,0	8,0	2569	− 66,5	− 9,8
L. Berger	44,00940	72,53140	0,5	125	27	Open	–	9,7	8,8	2938	− 60,7	− 9,7
Toro (Coy)A	45,53142	71,85494	0,0	717	19	Open	–	1,1	8,5	807	− 64,9	− 4,9

Additionally, Supplementary Information Table S1 contained formatting errors. The original Table S1 and accompanying legends appear below.

**Table S1:** Lakes with 2+ sampling years

Lakes with 2 or more sampling years							
Average 1σ δ^2^H (‰)	Average 1σ δ^18^O (‰)						
2.8	0.9						

	Number of samples (n)	Avg. δ^2^H (‰) VSMOW	Avg. δ^18^O (‰) VSMOW	Latitude (°S)	Longitude (°W)	Mult-year 1σ δ^2^H (‰)	Mult-year 1σ δ^18^O (‰)
Captren	2	− 64.4	− 9.3	− 38.6	− 71.7	0.2	0.1
Conguillo	2	− 71.7	− 9.5	− 38.6	− 71.6	1.2	0.2
El Toro	3	− 64.9	− 4.9	− 40.8	− 72.3	8.9	2.4
Galletue	2	− 76.7	− 10.2	− 38.7	− 71.3	1.3	0.1
Malleco	2	− 64.5	− 9.4	− 38.2	− 71.8	0.6	0.3
Negra	3	− 59.5	− 7.1	− 38.6	− 71.8	8.1	2.4
Quepe	2	− 58.5	− 6.4	− 38.6	− 71.9	2.2	1.2
San Pedro	2	− 61.9	− 5.3	− 38.4	− 71.3	5.6	1.6
Berger	2	− 65.8	− 8.9	− 44.0	− 72.5	2.7	0.8
Cajunco	2	− 33.3	− 4.6	− 42.2	− 73.8	1.0	0.0
Millan	2	− 34.4	− 4.0	− 42.6	− 73.8	2.7	0.9
NNTantrauco	2	− 38.1	− 5.5	− 43.0	− 73.8	3.7	0.1
Rinihue	2	− 32.5	− 3.6	− 42.8	− 73.9	2.4	1.8
Cirpreces	2	− 34.8	− 3.5	− 43.1	− 73.5	1.8	1.5
Verde Tolhuaca	2	− 66.7	− 9.2	− 38.2	− 71.7	0.4	0.4

The original Article and accompanying Supplementary Information files have been corrected.

