# Supplementary material for: Lake water based isoscape in central-south Chile reflects meteoric water
Source: Sci Rep. 2021 Apr 22;11:8725. doi: 10.1038/s41598-021-87566-4 (PMC8062518; doi:10.1038/s41598-021-87566-4)
Supplement: Supplementary file 2 — Supplementary Figures. [file 41598_2021_87566_MOESM2_ESM.docx]

Lake water based isoscape in central-south Chile reflects meteoric water

**Wesley P. Scott^1^, Sergio Contreras^*1,2^, Gabriel J. Bowen^3^, T. Elliott Arnold^1^, Ramón Bustamante-Ortega^4^, Josef P. Werne^1^**

^1^Department of Geology and Environmental Science, University of Pittsburgh, Pittsburgh, Pennsylvania, USA. [wps17@pitt.edu](mailto:wps17@pitt.edu), [jwerne@pitt.edu](mailto:jwerne@pitt.edu), [tea27@pitt.edu](mailto:tea27@pitt.edu)

^2^Departamento de Quiímica Ambiental, Facultad de Ciencias & Centro de Investigación en Biodiversidad y Ambientes Sustentables (CIBAS), Universidad Católica de la Santísima Concepción, Casilla 297, Concepción, Chile. [scontreras@ucsc.cl](mailto:scontreras@ucsc.cl)

^3^Department of Geology and Geophysics, University of Utah, Salt Lake City, Utah, USA. [gabe.bowen@utah.edu](mailto:gabe.bowen@utah.edu)

^4^Centro de Información de Recursos Naturales (CIREN), Santiago, Chile. [rbustamante@ciren.cl](mailto:Bustamante.ortega@gmail.com)

***Correspondence:**Sergio Contreras
[scontreras@ucsc.cl](mailto:scontreras@ucsc.cl)

**Keywords: Hydrogen, Oxygen, Deuterium excess, Isoscape, Lake water, Meteoric.**

Supplementary Information


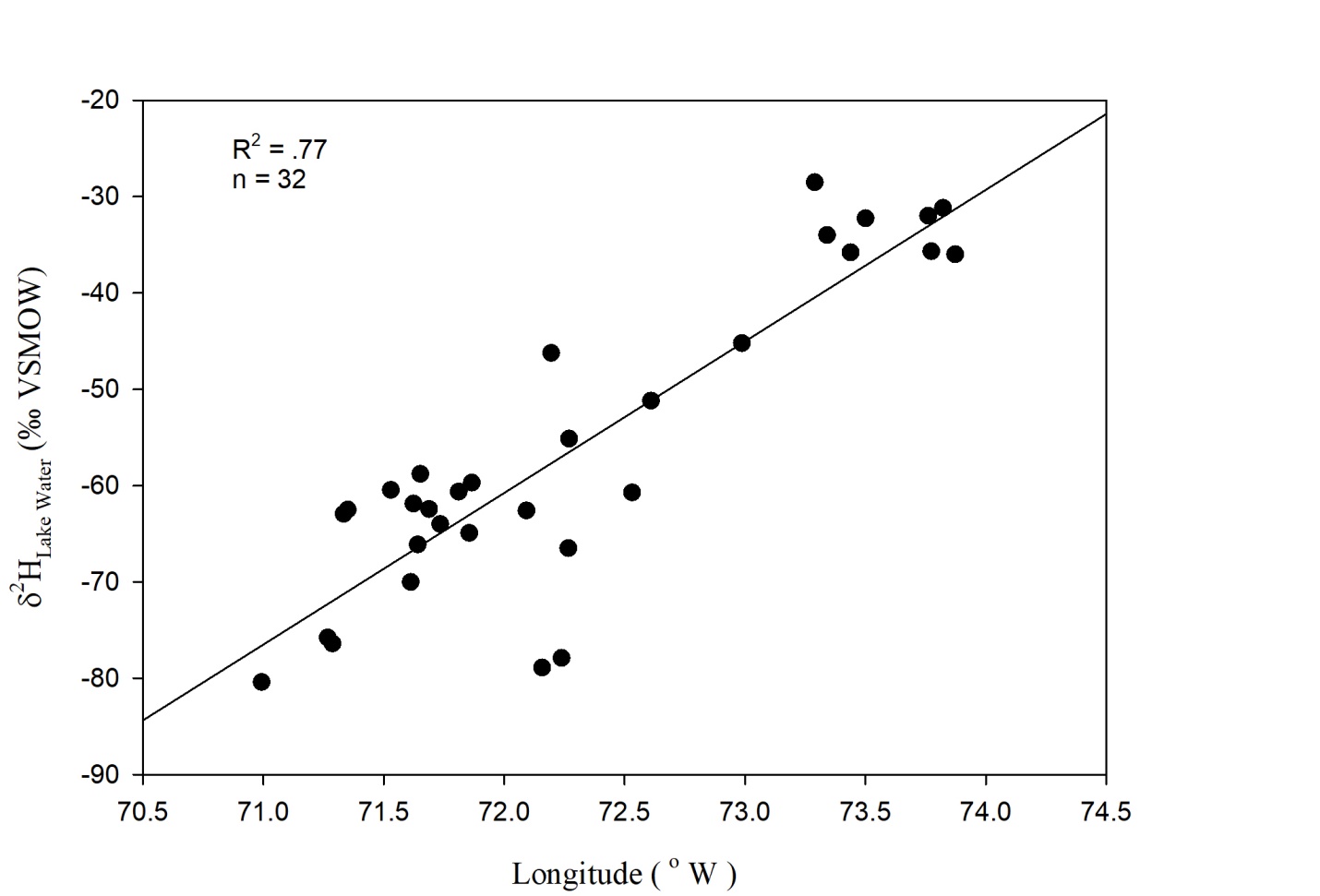


Supplementary figure 1. Plot of δ^2^H_Lake Water_ and longitude.


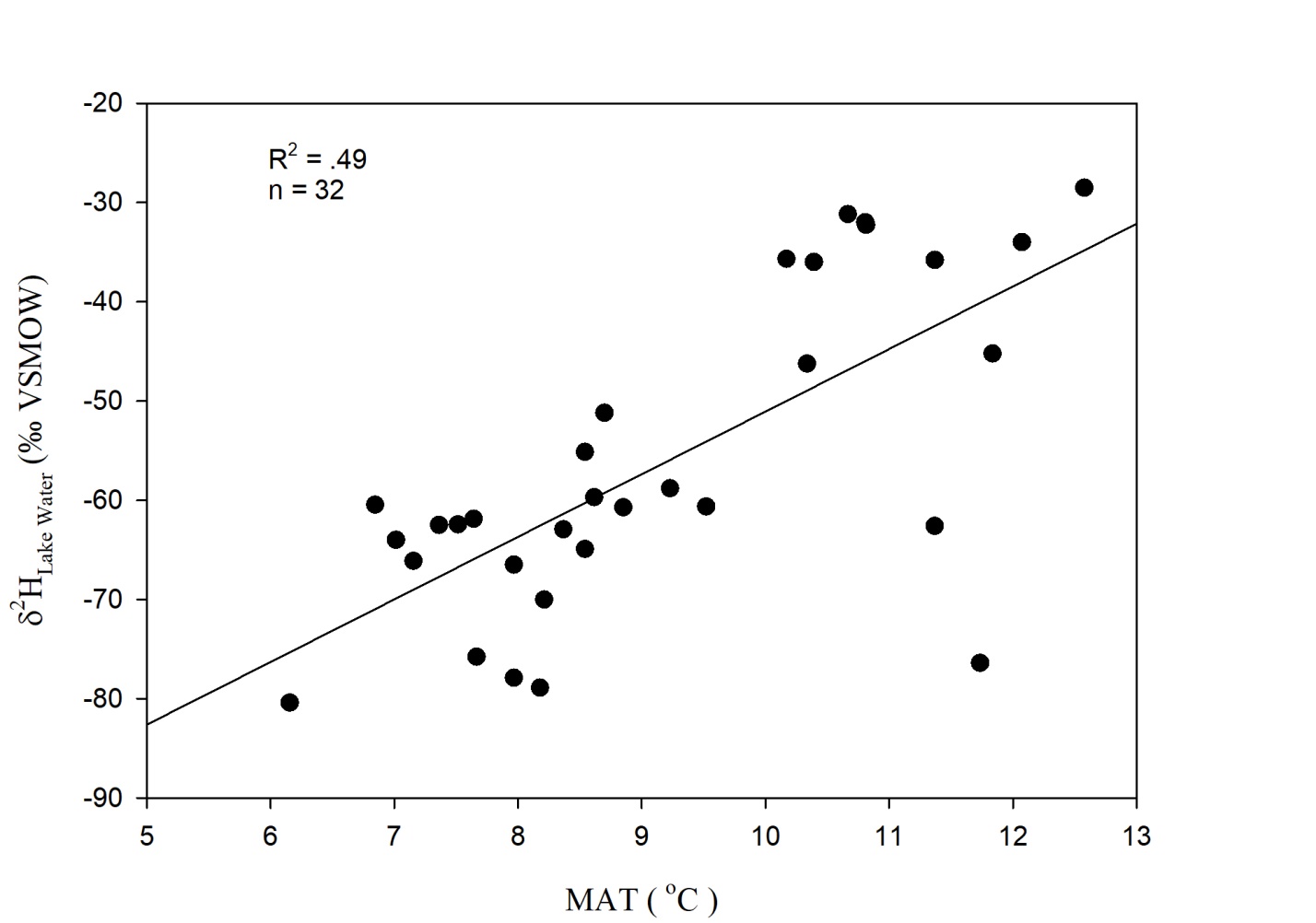


Supplementary figure 2. Plot of δ^2^H_Lake Water_ and and mean annual temperature (MAT).


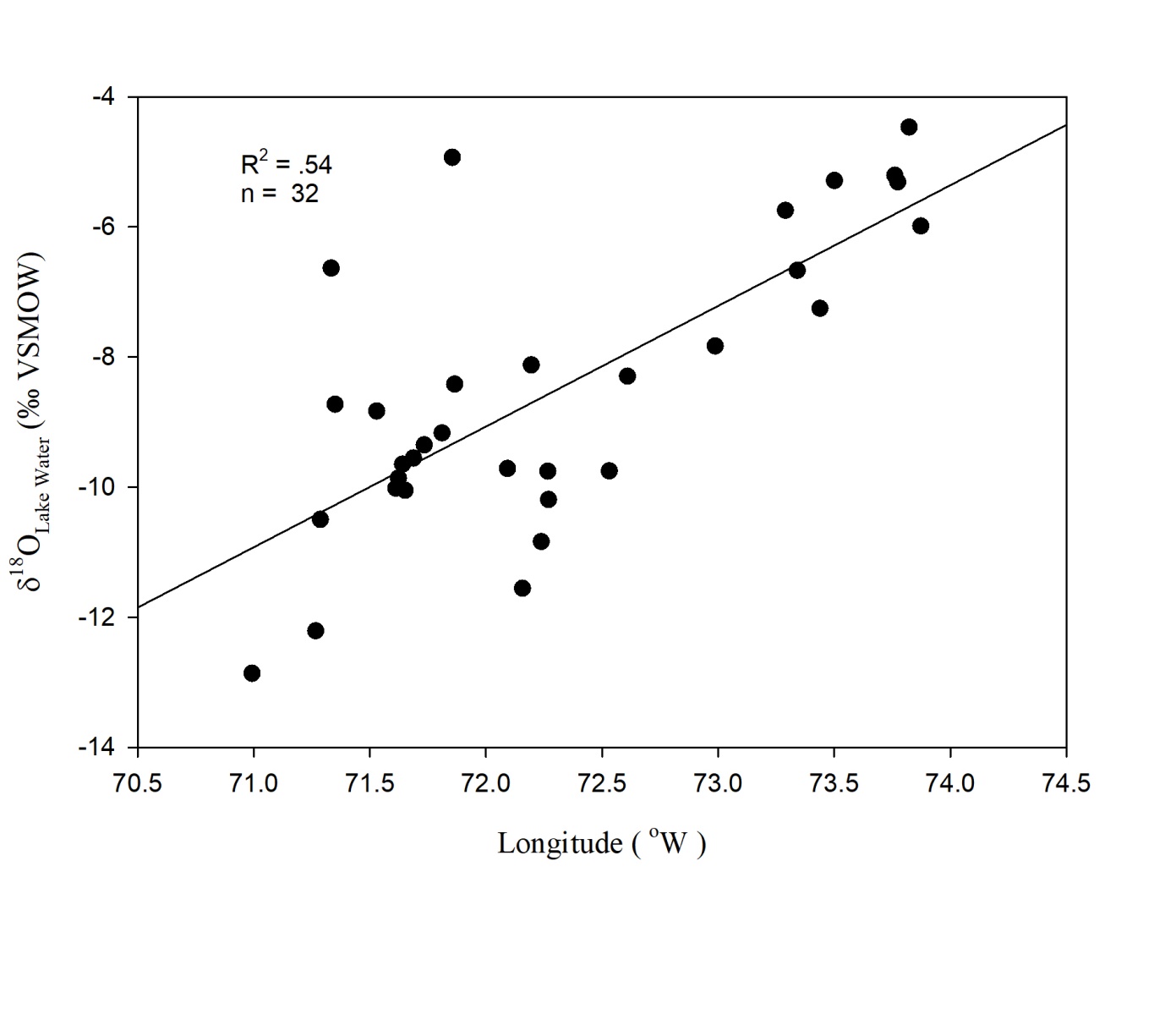


Supplementary figure 3. Plot of δ^18^O_Lake Water_ and and longitude.

Supplementary table 1. Lakes with at least 2 years of discrete and consecutive sampling. All sampling occurred between November and February (Austral Summer).

| **Lakes with 2 or more sampling years** |  |  |  |  |  |  |  |
| --- | --- | --- | --- | --- | --- | --- | --- |
| **Average 1σ δ2H (‰)** | **Average 1σ δ18O (‰)** |  |  |  |  |  |  |
| 2.8 | 0.9 |  |  |  |  |  |  |
|  | **Number of Samples (n)** | **Avg. δ2H (‰) VSMOW** | **Avg. δ18O (‰) VSMOW** | **Latitude (°S)** | **Longitude (°W)** | **Mult-year 1σ δ2H (‰)** | **Mult-year 1σ δ18O (‰)** |
| Captren | 2 | -64.4 | -9.3 | -38.6 | -71.7 | 0.2 | 0.1 |
| Conguillio | 2 | -71.7 | -9.5 | -38.6 | -71.6 | 1.2 | 0.2 |
| El Toro | 3 | -64.9 | -4.9 | -40.8 | -72.3 | 8.9 | 2.4 |
| Galletué | 2 | -76.7 | -10.2 | -38.7 | -71.3 | 1.3 | 0.1 |
| Malleco | 2 | -64.5 | -9.4 | -38.2 | -71.8 | 0.6 | 0.3 |
| Negra | 3 | -59.5 | -7.1 | -38.6 | -71.8 | 8.1 | 2.4 |
| Quepe | 2 | -58.5 | -6.4 | -38.6 | -71.9 | 2.2 | 1.2 |
| San Pedro | 2 | -61.9 | -5.3 | -38.4 | -71.3 | 5.6 | 1.6 |
| Berger | 2 | -65.8 | -8.9 | -44.0 | -72.5 | 2.7 | 0.8 |
| Cajunco | 2 | -33.3 | -4.6 | -42.2 | -73.8 | 1.0 | 0.0 |
| Millan | 2 | -34.4 | -4.0 | -42.6 | -73.8 | 2.7 | 0.9 |
| NN Tantauco | 2 | -38.1 | -5.5 | -43.0 | -73.8 | 3.7 | 0.1 |
| Rinihue | 2 | -32.5 | -3.6 | -42.8 | -73.9 | 2.4 | 1.8 |
| Cirpreces | 2 | -34.8 | -3.5 | -43.1 | -73.5 | 1.8 | 1.5 |
| Verde Tolhuaca | 2 | -66.7 | -9.2 | -38.2 | -71.7 | 0.4 | 0.4 |
